# Exploring medication experiences, behaviours, and reminder technology preferences in people with Parkinson's disease: a survey

**DOI:** 10.3389/fdgth.2026.1839031

**Published:** 2026-07-30

**Authors:** Emma Packer, Héloïse Debelle, Annette Hand, Lynn Rochester, Alison J. Yarnall, Lisa Alcock, Silvia Del Din

**Affiliations:** 1 Translational and Clinical Research Institute, Faculty of Medical Sciences, Newcastle University, Newcastle upon Tyne, United Kingdom; 2 Research Institute for Sport and Exercise Sciences, Liverpool John Moores University, Liverpool, United Kingdom; 3 School of Healthcare and Nursing Science, Northumbria University, Newcastle upon Tyne, United Kingdom; 4 The Newcastle Upon Tyne Hospitals NHS Foundation Trust, Newcastle upon Tyne, United Kingdom; 5 National Institute for Health and Care Research (NIHR) Newcastle Biomedical Research Centre (BRC), Newcastle University, Campus for Ageing and Vitality, Newcastle upon Tyne, United Kingdom

**Keywords:** adherence, medication, neurodegeneration, Parkinson's, wearables

## Abstract

**Introduction:**

Due to complex medication regimens, suboptimal medication adherence is common among people with Parkinson’s disease (PwP), leading to poor symptom control. Medication reminder technology, and a deeper understanding of individual’s medication experiences and behaviours offer promising solutions, but these factors need to be explored in PwP. An online survey was developed to identify the current potential use of medication reminder technology in PwP; determine the essential features of the ideal medication reminder technology; compare feature preferences between current medication reminder technology users and non-users; and Explore Respondents Medication Experiences and Behaviour.

**Methods:**

The survey was open to individuals over 18, with a PD diagnosis and taking oral PD medication. The survey consisted of 51 primary questions exploring PwP medication experiences and behaviours; current/potential use of medication reminder technology; and designing ideal medication reminder technology. Descriptive statistics, and statistical tests compared questionnaire responses. To categorise free-text responses, inductive and deductive content analysis was performed.

**Results:**

Data from 283 PwP (median age: 65 years, 46% female) was analysed. Thirty-four percent were non-adherent to their PD medication regimen, and respondents with more negative medication beliefs and perceived medication complexity had worse medication adherence. Fifty percent of respondents already used medication reminder technology, with these respondents most commonly using smartphones (72%). Respondents who already used medication reminder technology scored significantly higher on the medication adherence rating scale (MARS-5). When choosing the ideal medication reminder technology, smartwatches were the preferred device (57%). When designing the ideal medication reminder smartwatch, no features were deemed ‘essential’ to all respondents, with great heterogeneity in preferences. Overall, smartwatch usability and functionality was more important to respondents than appearance, and current users of reminder technology placed greater importance on smartwatch usability than non-users.

**Discussion:**

Medication reminder technology, such as smartwatches, may support medication adherence in PwP, but this technology must be personalised to user needs, and developed with an emphasis on usability and functionality, over aesthetics. When developing this technology, PwP should be included as co-developers, and the process should be iterative. However, reminder technology alone may not sufficiently support adherence, and additional patient-related factors need to be overcome.

## 1 Introduction

At present, there are no disease modifying treatments for Parkinson's disease (PD), with existing dopaminergic medications providing only symptomatic relief (1). Due to the progressive nature of PD, the complexity of medication regimens increases over time, with effective symptom management reliant on more frequent dosing and/or higher dosages, as well as strict adherence to prescribed regimens (2, 3). Due to this complexity, suboptimal medication adherence is common among people with PD (PwP), leading to poor symptom management and subsequent reduced quality of life (QoL) (4). Digital health technology offers a promising solution, with technology such as smartwatches able to provide audible/vibrating medication reminders, potentially improving medication adherence and symptom control in PwP (5, 6).

In previous research we explored the usability and feasibility of a digital health technology system to monitor mobility through a body-worn sensor and provide medication reminders using a smartwatch and smartphone in PwP (7). This research highlighted that in PwP, one style of smartwatch does not suit all, with participants experiencing a variety of challenges using the same smartwatch, and reporting different preferences on smartwatch features, appearance, size, and notification delivery preferences (auditory and vibration) (7). This research also concurred with previous literature in PwP, observing that wrist-worn devices were preferable to handheld devices such as smartphones (8).

As technology which provides medication reminders has the potential to support medication adherence, symptom control, and QoL in PwP, it is essential that the needs and expectations for this technology are explored with end users. It is also important to understand whether technology preferences differ between individuals who already use medication reminder technology and those who do not. This will ensure that observations are more generalisable and do not disproportionally represent those who do not currently reminder technology, as these individuals are less likely to have experienced barriers associated with their use (e.g., their tremor making it more difficult to click the right button), and thus less likely to express preferences related to this.

At present, there is a paucity of literature which explores patient preference for technology before it is developed, with insights typically gained from feasibility and usability studies on preexisting devices (7, 9, 10). Identifying device preferences prior to development could aid in the co-design of the ideal medication reminder technology and support in the development of usable and acceptable technology for PwP (11). However, previous literature has observed significant associations between various factors (e.g., regimen complexity) (12) and worse medication adherence in PwP, meaning reminder technology alone, may not sufficiently support medication adherence. It is therefore essential to explore medication experiences and behaviours in PwP, and how these associate with medication adherence and current/future technology preferences. To obtain these critical insights we developed an online survey for PwP, which aimed to:

- Explore medication experiences and behaviours in PwP,
- Identify current and potential use of medication reminder technology in PwP,
- Determine the essential features of the ideal medication reminder technology for PwP,
- Compare ideal technology preferences between current medication reminder technology users and non-users

## 2 Materials and methods

To achieve study aims we developed an online survey, which was available between 7th February – 30th September 2023. The open survey was anonymous, and available internationally to individuals over 18, with a PD diagnosis, and taking oral PD medication. Individuals not meeting this criterion were excluded from taking part. The survey was developed and published in English, and survey questions are provided in appendix 1. Survey reporting follows the Checklist for Reporting Results of Internet E-Surveys (CHERRIES) (Supplementary Table S6) – to ensure ease of reading, the majority of this information is provided in Supplementary Material (13).

### 2.1 Ethics

The survey received ethical approval from the Newcastle University Ethics Committee (Ref: 27681/2022) and was part of the larger, Medical Research Council (MRC) Confidence in Concept (CiC) funded study “Translating digital healthcare to enhance clinical management: evaluating the effect of medication on mobility in people with Parkinson’s Disease” (ISRCTN: 13156149, https://www.isrctn.com/ISRCTN13156149).

### 2.2 Survey development and recruitment

The survey was designed with input from PD experts, including specialist clinicians, physiotherapists, human movement scientists, and clinical researchers. A convenience sampling method was utilised. To recruit participants, a digital and paper flyer was developed and distributed through social media platforms (X and LinkedIn), shared at local PD support groups, and made available to the public. PD-focused charities promoted the survey through mailing lists; for example, Parkinson's UK included a study summary in their “Take part in research” emails. In addition, a dedicated post outlining the survey and participation details was published on the Parkinson's North East and Cumbria Research Interest Group (NEC-RIG) website, and Parkinson's Europe shared survey information on their X account. Parkinson's researchers and clinicians from across the world were also contacted via email and were invited to distribute the survey to their network.

### 2.3 Questions

The survey consisted of 51 primary questions made up of demographic questions and dichotomous (yes/no) questions, free-text responses, Likert scales, multiple choice, and validated/custom questionnaires. Visitors entering the survey read the survey information and provided informed consent, by clicking ‘Yes, I would like to complete this survey’. Conditional questions were included based on respondents’ answers, allowing those who responded in a certain way, to provide more information. Respondents were able to choose or type ‘prefer not to say’ to all questions but the validated questionnaires – Medication Adherence Rating Scale (MARS-5) (14) (©Professor Rob Horne), Parkinson's Disease Quality of Life questionnaire (PDQ-8) (15), and medication beliefs scale (PD-Rx) (16). To minimise misunderstanding and avoid misclassifying responses from open text questions, any answers which did not fit into predefined categories were reported exactly as written.

#### 2.3.1 Clinical and demographic

Respondents reported their age, sex, country and region of residence, postcode, living arrangements, occupational status, volunteering and physical activities, walking aid use, any comorbidities, years since diagnosis, if they had deep brain stimulation, their PD symptoms, and completed the validated PDQ-8 (15, 17) (license granted by Oxford University Innovation). Respondents used a Likert scale to report the intensity and frequency of each symptom in the seven-days prior to completing the survey.

#### 2.3.2 PD medication

Respondents reported the name, dosage, and intake times of their PD medications, and if they received any helping taking them, or had any ‘top-up’ (as needed, PRN (pro re nata)) medication. Respondents reported when they last received a medication review and highlighted if they had received enough information from their PD specialist about the importance of taking their medication as prescribed, and if not, which information they would have liked to receive.

#### 2.3.3 Medication beliefs, complexity, adherence

To determine medication beliefs, respondents completed the Parkinson's disease ‘Medication Beliefs Scale’ (PD-Rx) (16), with respondents rating their agreement to 11 statements (scored 11–55) on a 5-point Likert Scale - higher scores, suggestive of more positive beliefs. Authorisation to use the PD-Rx was granted by authors. To determine individual's perceived medication complexity, respondents rated complexity on a 5-point Likert scale, ranging from ‘not at all complex’ to ‘extremely complex’. To determine PD medication adherence, respondents completed the Medication Adherence Rating Scale (MARS-5) (14) in which respondents answered five questions on a Likert scale from ‘Always’ (1) to ‘Never’ (5) with scores 22 or below deemed non-adherent, and scores ranging from 5 to 25 (18).

#### 2.3.4 PD medication effect, side effects, and symptom fluctuations

Respondents reported: how their symptoms changed following PD medication intake; medication side effects in the seven days prior to completing the survey, and ever; how often they had experienced dyskinesia and motor symptom fluctuations in the seven days prior to completing the survey; and highlighted if dyskinesia and fluctuations stopped them from completing their daily activities and whether avoiding them was important to them.

#### 2.3.5 Current and potential use of medication reminder technology

Respondents reported which device (if any) they used to provide medication reminders, and were specifically asked if they used a smartwatch to provide medication reminders, and if so, which features they liked about it – choosing from a predefined list of 14 features, which were based on previous literature (7, 19). To identify potential medication reminder technology usage, respondents reported: which device they would be most and least likely to use for medication reminders; if they would wear a device which is visible to others or one that is easily concealed by clothing; if they would be happy for their smartwatch data to be used for other purposes, and if their clinician asked them to wear a smartwatch to provide medication reminders, would they, and for how long.

#### 2.3.6 Designing the ideal medication reminder smartwatch

Respondents highlighted which screen size their “perfect” smartwatch would have, from “small, medium or large” and justified their choice as a free text response. Respondents also reported which features the “perfect” smartwatch should have, rating their agreement to 11 smartwatch features on a five-point Likert scale from “strongly agree” to “strongly disagree”.

### 2.4 Data and statistical analysis

Only completed surveys were included in analysis. All analysis was performed using the Statistical Package for the Social Sciences (IBM SPPS, version 29). Descriptive statistics were used to describe quantitative responses. Quantitative data were checked for normality using the Shapiro–Wilk test, and visual inspection of histograms. Spearman's rank tests were performed to assess relationships between variables. Where appropriate, one-way ANOVAs and Kruskal–Wallis tests were used to compare means/medians between groups. To compare mean scores between independent groups Mann Whitney U tests were used. Fisher's Exact Tests were performed to explore associations between current reminder technology users and non-users, with the statistical threshold set to *p* < 0.05. Free-text responses were analysed using deductive and inductive content analysis (more detail in Supplementary Material).

## 3 Results

### 3.1 Demographic and clinical characteristics

A total of 301 PwP completed the survey. However, five duplicate responses were removed, with an additional two respondents excluded due to invalid responses (calling their medication ’sweeties’ or stating that they took one million tablets a day), leaving 294 respondents. Additionally, due to few responses from PwP outside the United Kingdom (UK), a further 11 respondents from countries outside the UK, were removed.

Data from 283 UK-based respondents was analysed (Table 1, Supplementary Table S1). Respondents had a median age of 65 years, and 45% were female. Median time since diagnosis was 4 years (range = 2 weeks to 30 years), and median QoL score (PDQ-8) was 22 (range = 0–81) (Supplementary Figure S1). Ninety-five percent of respondents lived in the community, 72% were retired, and 90% were physically active (Supplementary Figure S2).

**Table 1:** Demographic and clinical characteristics of survey respondents.

Characteristics	Median (min-max)	Frequency
Sex: n, %		Female: 127 (45%), Male: 156 (55%)
Age (years)	65 (37–89)	<40: *n* = 2
		40–50: *n* = 17
		51–60: *n* = 72
		61–70: *n* = 100
		71–80: *n* = 86
		81+: *n* = 6
Time since diagnosis	4 (2 weeks – 30 years)	
Country of residence		England: 86.6%
		Scotland: 7.4%
		Wales: 3.5%
		Northern Ireland: 2.5%
Walking Aid		Yes: 21%, No: 79%
Occupational Status^a^		Working full-time: 12%
		Working part-time: 12%
		Retired: 72%
		Unemployed: 4%
Physically Active		Yes: 90%, No: 10%
Living arrangements		Community dwelling, living with someone: 82%
		Community-dwelling, living alone: 13%
		Living alone within supported living: 2%
		Living with someone in supported living: 0.4%
		Other: 3%
PDQ-8 Score	22 (0–81)	
PD Symptoms (In the seven days prior to completing survey, excluding ‘other’ symptoms)^b^	4 (1–9)	Bradykinesia: 78%
		Fatigue: 69%
		Tremor: 66%
		Pain: 51%
		Balance problems: 43%
		Wearing Off: 42%
		Speech Problems: 30%
		Freezing of Gait: 18%
Comorbidities^c^	1 (0–5)	76 different comorbidities reported
		Arthritis: 18%
		Hypertension: 14%
		Asthma: 9%
		Diabetes (I and II): 8%
		Hypothyroidism: 7%

a One respondent did not report their occupational status, and one respondent reported working 50 h in a ‘part-time’ role, and was excluded from this question.

b Median symptom number was calculated without including ‘other’ symptoms as 12 of 34 respondents who reported experiencing “other” symptoms did not report them.

c Twelve respondents provided insufficient detail to categorize and include their comorbidity.

In the seven days prior to completing the survey, respondents experienced a median of 4 (range = 1–9) of the most common PD related symptoms, with bradykinesia (defined as slowness of movement in the survey) most commonly experienced (78%) (Table 1). 26 ‘other’ PD symptoms were reported, with dystonia, cramps, and constipation most commonly reported (*n* = 4 each) (Supplementary Figure S3). A strong, significant positive correlation was identified between symptom frequency and intensity (*ρ*=0.952, *p* < 0.001) (Supplementary Figure S4, S5).

Fifty-two percent (*n* = 149) of respondents reported at least one comorbidity, however only 137 respondents provided sufficient detail to include responses in this question. Seventy-six different comorbidities were reported, with arthritis most commonly reported (18% of those with a comorbidity), followed by hypertension (14%) (Supplementary Table S2).

### 3.2 Medication insights, review, and complexity

Respondents took a median of six PD tablets per day (range = 1 to 19), across a median of four timepoints in the day (range = 1 to 9), most commonly at 8:00am (Supplementary Figure S6). Levodopa was most frequently taken (93%), with six respondents taking Anticholinergic medication, specifically Trihexyphenidyl (Table 2). Four respondents had deep brain stimulation and 29% (*n* = 83) were prescribed PRN (pro re nata, as needed) medication.

**Table 2:** Percentage of respondents taking each PD medication (only includes those who reported, *n* = 275). Percentage of respondents and time since their last medication review. Number of respondents that reported their medication regimen complexity and the number of medications they took.

Medication type^a^		Percentage (n)
Levodopa		93% (257)
Dopamine Agonist		31% (86)
Monoaminoxidase-B inhibitor (MAO-B I)		29% (80)
Catechol-O-methyltransferase inhibitor (COMT-I)		7% (19)
Combination Levodopa and COMT-I		6% (16)
Amantadine		6% (16)
Anticholinergic		2% (6)

Time since last Parkinson's medication review		Percentage (n)
Within the last month		14% (39)
Within the last 3 months		31% (88)
Within the last 6 months		30% (86)
Within the last year		19% (54)
Within the last 2 years		4% (12)
Over 2 years ago		1% (4)

Medication complexity	Median number of PD tablets/day (min-max)	Percentage (n)
Not at all complex	5 (1–17)	49% (139)
Slightly complex	6.5 (3–19)	27% (75)
Moderately complex	7 (2–19)	18% (52)
Very complex	8 (4–11)	5% (14)
Extremely complex	10 (3–19)	1% (3)

a Eight respondents did not report their PD medication and were not included in this table

Ninety-four percent of respondents had a medication review within twelve months of completing the survey (Table 2), whereas four respondents had not received a review for over two years. One of these four respondents took 18 tablets across six time points each day and highlighted that their PD specialist had not given them enough information about taking their medication, reporting they had “tweaked things myself and kept a journal for cause and effect”, and experienced side effects of “gambling and shopping addiction”. There was no significant difference in median time since diagnosis and the time since last PD medication review categories (*p* = 0.753).

Forty-nine percent of respondents felt their PD medication regimen was ‘not at all complex’, with 6% reporting it to be ‘very’ or ‘extremely’ complex (Table 2). There was a statistically significant increase in the median number of PD tablets per day with increased medication complexity (*p* < 0.001).

#### 3.2.1 Medication beliefs, support, and importance

Respondents’ median medication beliefs score (PD-Rx) was 37, ranging from 20 to 50 (Supplementary Figure S7). Overall, respondents had more positive beliefs about their medication, than negative beliefs, with 79% agreeing or strongly agreeing that their PD medication helps them live a more normal life, and gives them some control over their symptoms (Supplementary Figure S7). Medication belief scores were significantly negatively correlated with QoL scores (PDQ-8) (*ρ* = −0.185, *p* = 0.002), indicating that those with more positive medication beliefs may have a better QoL.

Fifteen percent (*n* = 43) of respondents received support with their PD medication. Specifically, 84% received support from their spouse, 5% from a carer, 2% from friends and family, and 2% from a partner and family. One respondent reported receiving support by ‘keeping a book’ but did not provide further detail. There was no significant difference in medication adherence scores (MARS-5) between those who received support when taking their medication and those who did not (*p* = 0.081).

Thirteen percent (*n* = 38) of respondents felt they had not received enough information from their clinician on the importance of taking their medication as prescribed. Of these respondents, 15 highlighted the information they would have liked to receive. Respondents most commonly wanted more information on medication timings (*n* = 12), including “best timings of medication”, whether medication can be taken “5…15…or 30 min” late/early, and at “what intervals … pre and post food” medication can be taken. Secondly, respondents required more information on medication effects (*n* = 6), specifically “the impact of forgetting to take medication” or taking more medication, and the “side effects”. Lastly, respondents (*n* = 4) wanted more information about the influence of food, for example, “avoiding certain foods” such as “protein” and whether to take “on an empty stomach” (Supplementary Table S3).

#### 3.2.2 Medication effect

Following PD medication intake, 25% (*n* = 72) of respondents reported that they typically experience ‘fewer symptoms until the next medication dose’; 30% (*n* = 85) typically experience ‘fewer symptoms for some time’, which return before their text medication dose; 26% (*n* = 73) typically experience fluctuating symptoms; and 19% (*n* = 53) find that their symptoms do not change following medication intake.

**Figure 1:**
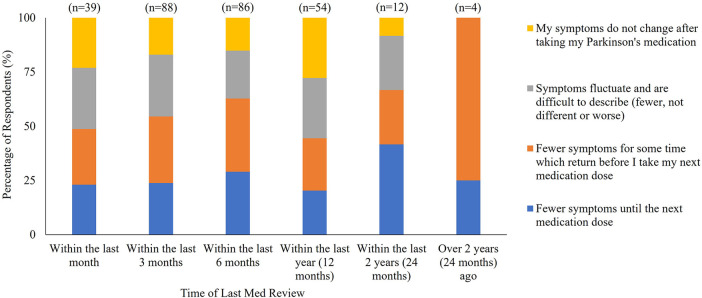
Percentage of respondents within each time period since their last medication review and the effect they perceive their medication to have on symptoms.

Among respondents who received a medication review within one, three, or six months prior to completing the survey, there was an equal proportion of respondents within each medication effect category (Figure 1). There was a 13% increase in the number of respondents who felt their symptoms did not change following medication intake from six to twelve months since attending a medication review. Three of the four respondents who had not received a medication review within the last two years felt their symptoms returned before taking their next medication dose (wearing off) (Figure 1). Medication side effects are discussed in section 2.8 of Supplementary Material.

#### 3.2.3 Medication adherence

**Figure 2:**
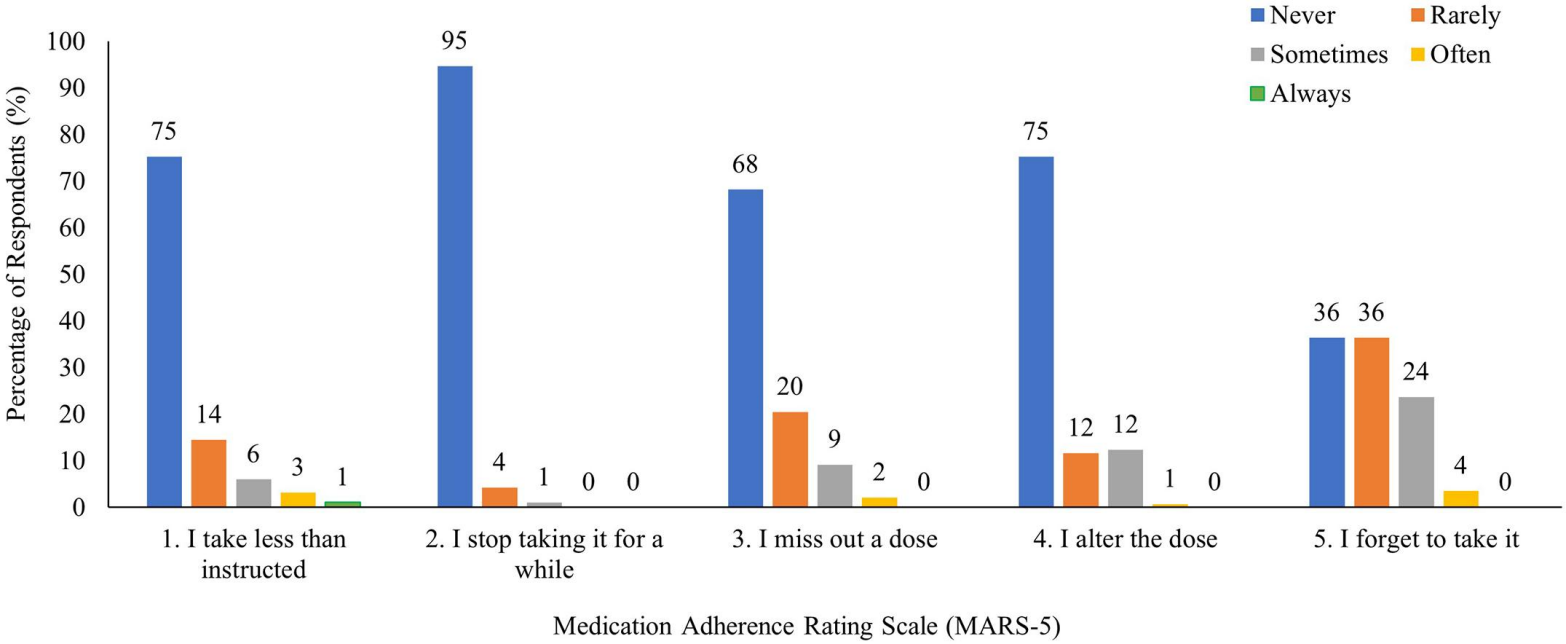
Percentage of respondents and their responses to each question of the medication adherence scale (MARS-5).

Thirty-four percent of respondents were classified as non-adherent to their PD medication regimen, scoring 22 or lower on the medication adherence rating scale (MARS-5) (Figure 2). Specifically, 28% of respondents reported that they often or sometimes forget to take their PD medication. Twelve percent ’sometimes’ altered their dose, and 9% ’sometimes’ missed out a dose, whilst 6% percent sometimes took ‘less than instructed’ (Figure 2).

Medication adherence scores (MARS-5) were significantly negatively correlated with the number of PD tablets per day (*ρ* = - 0.226, *p* < 0.001), and QoL score (PDQ-8) (*ρ* = −0.209, *p* < 0.001). This suggests that respondents who took more PD tablets per day and had worse QoL were less adherent to their prescribed regimen – although the direction of this relationship cannot be determined from this analysis. Medication adherence scores were significantly positively correlated with medication belief scores (PD-Rx) (*ρ* = 0.231, *p* < 0.001), suggesting that individuals with more positive beliefs (higher PD-Rx score) may have greater medication adherence. Additionally, there was a statistically significant difference in medication adherence scores across different medication complexity levels (*p* = 0.002), with those reporting their medication regimen to be ‘not at all complex’ scoring significantly higher adherence scores than those reporting a ‘moderate’ or ‘very’ complex regimen. There was no significant difference in medication adherence scores between respondents in each category of perceived medication effect (e.g., symptoms improve, symptoms stay the same etc).

### 3.3 Medication reminder technology

#### 3.3.1 Current use of technology for medication reminders

Fifty percent (*n* = 142) of respondents already used a digital device to remind them to take their PD medication, whilst 41% reported that they would use one, 4% would not use one, and 5% were unsure. Of the respondents who already used medication reminder technology, 72% (*n* = 102) used smartphones, and 26% (*n* = 37) used smartwatches. Other common medication reminder methods included: an application or alarm on an unspecified device (*n* = 11), alarmed pill box (*n* = 4), an Alexa, tablet (e.g., iPad), cooker timer, CUE1 device, or a computer. Reasons for not wanting to use a digital device included: not feeling the need (*n* = 6) (average age = 67.5), already using a pill box, not following a set medication regimen (*n* = 2), (*n* = 1) preferring to remember themselves, privacy concerns, independence concerns, not liking to use technology, and digital literacy issues (Supplementary Table S5).

Only medication adherence scores (MARS-5) were significantly different (higher) among respondents who already used medication reminder technology compared to those who did not (answered: I would use a device, I don't know, and no) (z = −2.771, *p* = 0.006). Whilst there was no significant difference in the age, number of tablets per day, time since diagnosis, QoL score, or the number of comorbidities between those who already used reminder technology and those who did not (*p* > 0.05).

Respondents were specifically asked if they used a smartwatch for medication reminders, with twenty-three percent (*n* = 66) already using one, with the majority liking that their smartwatch vibrated to provide medication reminders (86%), that it did not interfere with their daily life (79%), and charges easily (68%) (Table 3).

**Table 3:** Percentage of respondents who liked each feature of their current smartwatch.

Features of current smartwatch	Percentage of respondents who liked this feature
It vibrates to indicate my medication time	86%
It does not interfere with my daily life	79%
It charges easily	68%
It is easy to program	56%
It is waterproof	50%
It has an auditory alarm to indicate my medication time	48%
The screen lights up to indicate my medication time	41%
The battery does not need to be charged too often	36%
It looks nice	35%
It attaches easily	33%
The screen is big	29%
It is sturdy	26%
I can conceal it in my clothes	24%
The screen is small	11%

#### 3.3.2 Potential use of technology for medication reminders

Of the suggested devices to provide medication reminders, respondents were most likely to want to use a smartwatch (57%), and least likely to want to use a talking clock (48%) (Table 4). Eighty-one percent and 89% of respondents reported that they would wear medication reminder technology which is visible to others, or easily concealed by their clothing, respectively. Additionally, 94% of respondents were willing for any data collected by the technology to be used for other purposes (e.g., for research, or to help PD specialists understand symptoms). Moreover, 47% would be willing to charge the devices’ battery every night, and 17% would be willing to charge it two-three times per week, with two respondents choosing ‘prefer not to say’.

**Table 4:** Percentage and number of respondents who were most likely and least likely to use each device to help remind them to take their PD medication.

Device	Most likely to use % (n)^a^	Least likely to use % (n)^b^
Smartwatch or wrist band	57% (161)	3% (9)
Smartphone	33% (94)	6% (17)
Automated tablet dispenser with alarm	5% (15)	29% (83)
Digital calendar	2% (6)	11% (31)
Talking Clock	0% (1)	48% (137)
Other	1% (4)	0% (0)

a Two respondents chose “prefer not to say”.

b six respondents chose “prefer not to say”.

**Figure 3:**
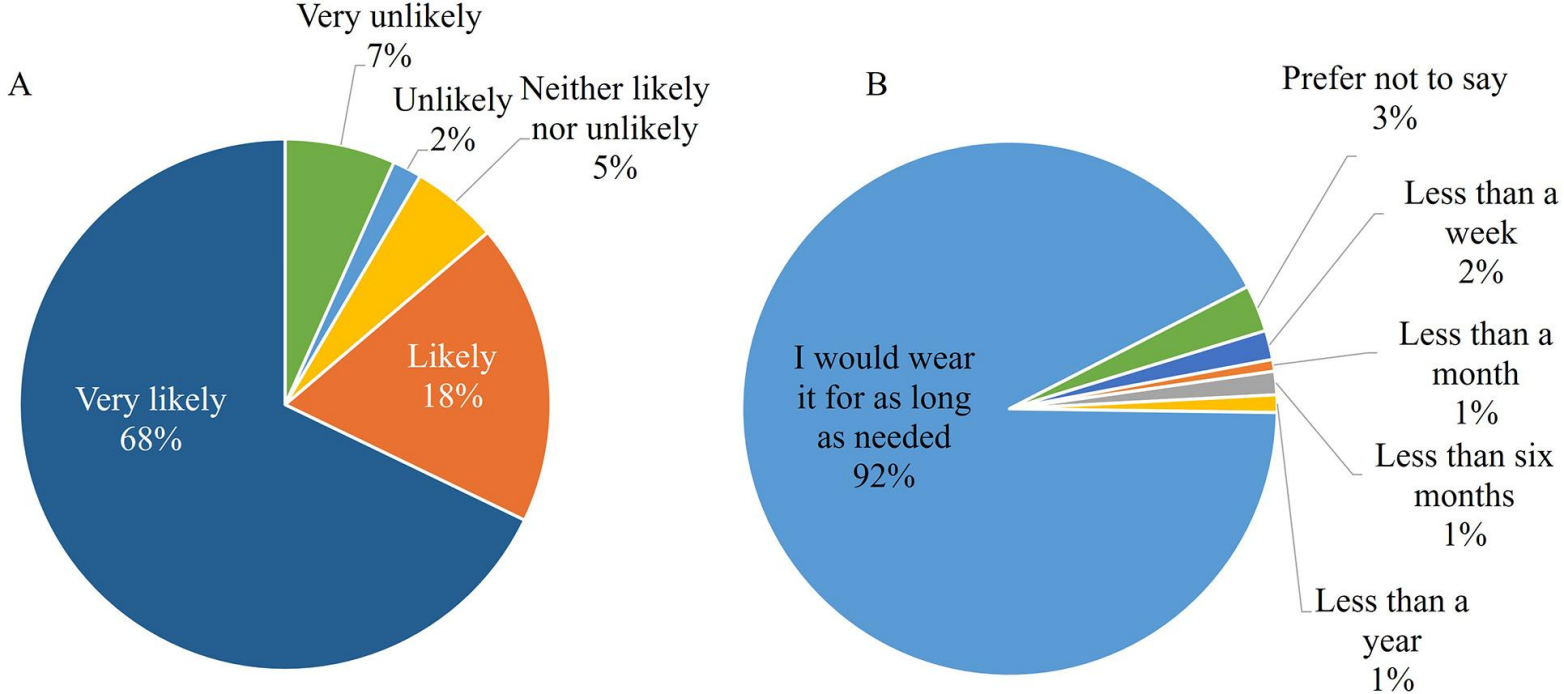
**(A)** percentage of respondents and their likelihood to wear a smartwatch for medication reminders, if their PD specialist asked them to wear one. **(B)** Percentage of respondents and the amount of time they would be willing to wear a smartwatch for if their clinician asked them to wear one. Eight respondents chose “prefer not to say”.

Following the smartwatch being the overall preferred device to provide reminders, if their clinician asked them to wear a smartwatch for medication reminders, 86% of respondents were very likely or likely to do so (Figure 3A), with 92% willing to wear the smartwatch for as long as needed (Figure 3B).

##### 3.3.2.1 Designing the ideal smartwatch for medication reminders

**Figure 4:**
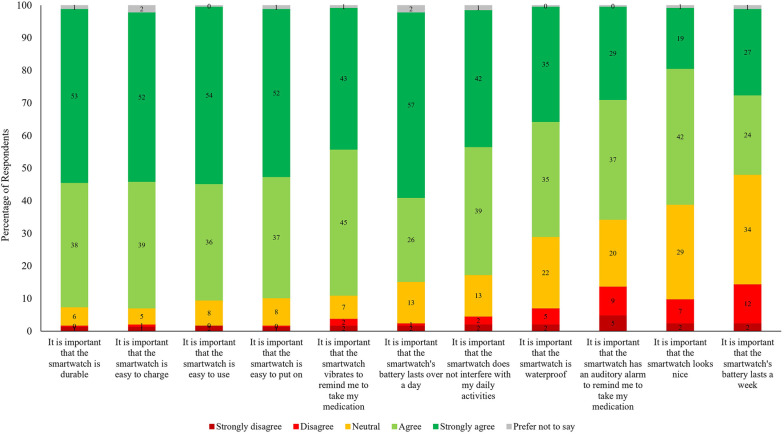
Percentage of respondents and their agreement to the ‘essential’ features of their ideal medication reminder smartwatch.

Of the 14 smartwatch features presented to respondents, no feature was considered ‘essential’ (agree) to the to all respondents. Durability and ease of charging were the top preferences, with 91% ‘agreeing’ or ’strongly agreeing’ with these statements. Similarly, 90% and 89% agreed or strongly agreed that the smartwatch needs to be ‘easy to use’ and ‘easy to put on’ respectively. Eighty-one percent agreed or strongly agreed that the smartwatch should not interfere with their activities of daily living, whilst 38% felt neutral or disagreed that the smartwatch needed to look nice (Figure 4).

In terms of specific smartwatch characteristics, 88% agreed or strongly agreed that the smartwatch should vibrate to provide medication reminders, whereas respondents were less concerned about auditory medication reminders, with only 66% agreeing or strongly agreeing with this (Figure 4), and 14% disagreeing or strongly disagreeing with this statement.

Eighty-three percent of respondents agreed or strongly agreed that the battery should last longer than a day, with respondents less concerned that the battery lasts a week – 14% disagreed or strongly disagreed with the statement “It is important that the smartwatch's battery lasts a week”, and 34% felt neutral (Figure 4). Additionally, respondents were less concerned that the smartwatch was waterproof, with 29% feeling neutral or disagreeing with this statement.

##### 3.3.2.2 Current use of reminder technology and smartwatch preferences

When designing the ideal medication reminder smartwatch, a significant association was observed between respondents who already used medication reminder technology and their preference for specific smartwatch features. Specifically, respondents who already used medication reminder technology were more likely to strongly agree that the ideal smartwatch should provide vibrating (*p* < 0.001, Table 5) and auditory (*p* = 0.004) medication reminders; be easy to use (*p* = 0.015); easy to put on (*p* = 0.015); easy to charge (*p* = 0.043); and be durable (*p* = 0.024) (Table 5). Additionally, respondents who already used reminder technology were more likely to disagree that the ideal smartwatch needed to be waterproof (*p* = 0.004). There was no significant association between current reminder technology use, and preference for the ideal smartwatch to look nice, to have a battery which lasts over a day or week, or for the watch to not interfere with daily activities (*p* > 0.05).

**Table 5:** Percentage of respondents in each response category to the statement “If you were to use a smartwatch to help you monitor your Parkinson's and send you medication reminders, please indicate how strongly you agree with each statement.” This table is used to design the ideal/perfect medication reminder smartwatch.

Smartwatch feature	Current use of reminder technology	Percentage of respondents in each response category					
		Strongly agree (%)	Agree (%)	Neutral (%)	Disagree (%)	Strongly disagree (%)	Number of respondents (n)
1. Vibrating medication reminders	Yes	52.8	38.0	5.6	3.5	0	142
	No	34.5	52.5	8.6	0.7	3.6	139
2. Auditory medication reminders	Yes	36.6	38.7	14.1	7.7	2.8	142
	No	20.6	34.8	27.0	9.9	7.1	140
3. Easy to use	Yes	62.0	31.7	6.3	0	0	142
	No	47.1	40.0	9.3	0	3.6	140
4. Easy to put on	Yes	60.0	31.4	8.6	0	0	140
	No	44.3	43.6	8.6	0.7	2.9	140
5. Easy to charge	Yes	57.7	39.4	2.9	0	0	137
	No	48.6	40.0	7.1	1.4	2.9	140
6. Durable	Yes	60.7	32.9	6.4	0	0	140
	No	47.1	44.3	5.0	0.7	2.9	140
7. Waterproof	Yes	35.2	32.4	23.9	8.5	0	142
	No	35.7	38.6	20.0	1.4	4.3	140
8. Looks nice	Yes	23.2	40.8	26.8	7.7	1.4	142
	No	14.4	43.2	31.7	7.2	3.6	139
9. Battery lasts over a day	Yes	62.0	24.8	12.4	0.7	0	137
	No	54.3	27.9	13.6	0.7	3.6	140
10.Battery lasts over one week	Yes	29.3	25.7	31.4	12.1	1.4	140
	No	24.3	23.6	36.4	12.1	3.6	140
11. Does not interfere with daily activities	Yes	45.7	40.0	10.0	3.6	0.7	140
	No	39.6	39.6	15.8	1.4	3.6	139

When comparing respondents who already used medication reminder technology to those who did not, there was no significant association between respondent’s grouping and how willing they were to wear medication reminder technology that is visible to others (*p* = 0.056), wear a device that is easily concealed (*p* = 0.351), how often they were willing to charge the battery (*p* = 0.736), how long they would be willing to a wear a device for (*p* = 0.306), and whether they were willing for their data to be used for other purposes (*p* = 0.532).

##### 3.3.2.3 Ideal smartwatch screen size

When asked about the ideal smartwatch screen size, 55% of respondents chose medium, 13% chose small, and 11% chose large, with 20% expressing no preference (*n* = 58) (Table 6). One respondent highlighted that “there would need to be a choice of sizes for something to be universally suitable”.

**Table 6:** Percentage of respondents in each category for smartwatch screen size preference reason.

	Smartwatch screen size preference^a^		
Reasons for size preference^b^	Small, *n* = 36 (%)	Medium *n* = 156 (%)	Large *n* = 32 (%)
Vision	6	58	88
Unobtrusive	31	19	0
Like a typical watch/smartwatch	17	14	3
Wrist size	19	4	3
Discrete	39	8	0
Comfort	6	3	0
Ease of use	3	1	25
Condition related (PD or other)	0	3	6

a One respondent chose ‘prefer not to say’ to the question asking about screen size preference.

b When asked to justify screen size preference, 12 respondents did not provide a response, and 10 respondents were excluded as they did not provide enough information.

Vision was the most commonly cited reason for preferring a medium (58%) or large (88%) screen, with preferences for a smaller screen primarily driven by the desire for discretion (39%) and unobtrusiveness (31%). When asked to justify smartwatch screen size preference, only seven respondents mentioned their PD or other conditions, with four respondents reporting a preference for a medium or large watch face due to their tremor, and expressed the desire for “buttons” as these “can be used with a shaky finger”. Two respondents reported that “skin problems” and “achy joints” influenced their screen size preference.

Additionally, three respondents highlighted that the “smartwatch would have to do much more than just tell me to take a tablet for it to be useful,” with one preferring if to be “multipurpose so tracked my activity as well as my PD”. Additionally, two respondents highlighted that the smartwatch “must have buttons rather than touchscreen,” as “touch screens and tremors do not mix well […] clicky buttons rule”.

## 4 Discussion

To our knowledge, this is the first large-scale survey in PwP to explore medication experiences, behaviours, current/potential use of medication reminder technology, and identify individual's preferences for developing the ideal medication reminder technology. Findings align with previous literature, observing that suboptimal medication adherence is common in PwP, and that various modifiable factors negatively influence this. Findings suggest that PwP who already use medication reminder technology may have better medication adherence, and that smartwatches are the preferred device to provide reminders. Exploratory analysis suggests that when designing the ideal medication reminder technology, no features will suit the needs and preferences of all PwP, and that preferences may be driven by prior experience with reminder technology, with current users placing greater emphasis on technology usability than non-users. For reminder technology to support medication adherence, development cannot adopt a one-size-fits-all approach and should instead be personalised to individual user needs and preferences, with technology usability at the forefront of development.

### 4.1 Developing the ideal medication reminder smartwatch

Among survey respondents’ prevalence of medication non-adherence was high and consistent with previous literature (20, 21). Respondents who already used reminder technology scored significantly higher on the medication adherence rating scale (MARS-5), suggesting that reminder technology may support better adherence (22), and could be utilised to support more favourable medication response and symptom control.

Of the respondents who already used reminder technology, smartphone/smartwatch alarms and applications were most commonly used, but smartwatches were the favourable device to provide medication reminders in the future. However, many medication reminder applications are not currently available on smartwatches; their feasibility and usability is rarely tested, and applications often lack personalisation options. To overcome this and provide medication reminders in a more user-friendly manner, a medication reminder smartwatch could be developed. Development of such technology may also reduce caregiver burden associated with continually reminding individuals of medication intake times. However, it is important to note that although a smartwatch was the most preferable for many respondents, 33% of respondents reported that a smartphone would be preferable. Future work should therefore explore the optimisation of smartphone-based medication reminders, ensuring that the needs of all PwP are considered.

This survey observed that when designing the ideal medication reminder smartwatch, PwP prioritise smartwatch ease of use and functionality, over device appearance and inclusion of additional features such as waterproofing and auditory notifications. The observed importance of usability is consistent with previous literature and is an essential aspect to consider when developing smartwatch technology (19, 23). However, when respondents were asked to rate which smartwatch features were essential to them, no features were considered essential to all respondents – highlighting the importance of developing smartwatch technology that can be personalised to user preferences. For example, a greater proportion of respondents expressed a preference for vibrating medication reminders over auditory reminders, but there was still a relatively large proportion who felt auditory reminders were essential. It is therefore crucial that the preferences of the majority do not disproportionately influence decision-making at the expense of individuals with less common preferences. This could be achieved by developing a smartwatch that has various features that can be activated/deactivated based on user preference. Findings also highlighted that future smartwatch screens need to be available in a range of sizes, ensuring that poor vision does not negatively influence usability, but also size does not influence daily activities.

This survey also observed that respondents who already used reminder technology were more likely to express usability-based smartwatch preferences (e.g., the watch being easy to use, charge, put on, and be durable) than non-users. This finding may be a consequence of individuals who already use reminder technology being aware of the barriers associated with technology, and thus placing greater importance on smartwatch usability. Additionally, when asked to provide rationale for their ideal smartwatch screen size, very few respondents mentioned their PD, with poor vision often cited for size preference. Although ophthalmological disorders are common in PwP (24) and are likely to influence smartwatch usability, this finding may also reflect that 40% of respondents were not currently using reminder technology, and thus may not have experienced the negative impact PD symptoms can have on device usability. It is therefore essential that during the development of future reminder technology, devices are pilot tested with PwP who have a range of symptoms and severity, and that an iterative user-centred design is employed. Additionally, there was no significant difference in the clinical characteristics of current medication reminder technology users and non-users, suggesting that clinical factors may not drive technology use, but other factors such as availability of devices and support when using them.

Overall, this survey highlighted that to encourage future use of medication reminder smartwatches, devices need to be easy to use, charge, put on, and be durable – features which can be tested in usability studies, which the World Health Organisation highlight as an essential first stage when developing new technology (25). Respondents who reported PD symptoms, highlighted that “touch screens and tremors do not mix well […] clicky buttons rule”. The ideal smartwatch should therefore be available as a touchscreen device, with tactical buttons, allowing individuals at different stages of their medication routine (e.g., OFF state) and PD stage, to more easily interact with the device. It is essential that prior to developing smartwatch technology, PwP are included as co-developers, and clinicians and carers are frequently consulted. Furthermore, as observed across literature and highlighted by one respondent the “smartwatch would have to do much more than just tell me to take a tablet for it to be useful”. Therefore, developing a smartwatch with other capabilities such as monitoring motor symptoms (e.g., tremor), heart rate and falls, may be preferable. However, medication reminder technology alone may not sufficiently improve adherence, and other influencing factors should be considered.

### 4.2 Medication experiences and behaviours in people with Parkinson's

In line with previous literature, this survey observed that other factors were associated with medication adherence in PwP and should be addressed. For example, this survey observed an association between worse adherence and forgetfulness, higher numbers of tablets per day, negative medication beliefs, and perceived regimen complexity. As non-adherence is associated with poor symptom control and reduced QoL in this survey and previous literature (4), it is essential that factors which negatively influence adherence are improved. For example, treating mood disorders (20); utilising medication reminder technology (26); encouraging individuals to link their dose taking with everyday activities (20), and/or improving negative medication beliefs using adherence therapy which challenges negative medication beliefs (12). Moreover, prioritising improved medication education is also essential, as greater understanding helps individuals feel more involved in their care and empowered to manage their condition (27, 28). As highlighted by this survey and previous literature, this area may benefit from further attention, with 13% reporting that they had not received enough information about their medication from their clinician (29–31). It is essential that this information is reinforced at each appointment, and letters are sent to patient's homes to provide further reinforcement.

Contrary to previous research, the majority of respondents felt their PD medication regimen was ‘not at all’ or only ’slightly’ complex (4, 20, 32), with respondents who took the same number of tablets reporting complexity at both extremes, ‘not at all complex’ and ‘extremely complex’. This suggests that in PwP the number of PD-related tablets taken per day does not solely account for perceived medication complexity. Current medication adherence studies often rely on tablet count as a marker of regimen complexity, and may therefore benefit from considering patient-reported complexity in addition to the number of tablets taken.

Respondents who had more recently received a medication review (within 6 months of completing the survey) tended to report more stable and less fluctuating symptoms following medication intake, than respondents whose last medication review was within 12 months of completing the survey. This suggests an association between more recent medication reviews and greater reported symptom stability. As expected, more frequent medication reviews may aid symptom control and reduce fluctuations, suggesting that reviews every 6-months may be more beneficial than every 6–12 months (28, 31, 33). However, these results should be interpreted with caution, as due to the descriptive nature of this study, results do not support causal inferences and contextual factors such as the severity of symptoms prior to medication reviews cannot be accounted for. Due to the UK's ageing population, the demand for PD services is increasing but resources and clinicians are not, meaning more frequent appointments may not be plausible. To overcome this, and in line with the NHS Long Term Plan (34), emphasis needs to be placed on empowering patients to manage their condition. This could be achieved through healthcare initiatives, such as the ‘Home Based Care (HBC)’ pathway (28), which aims to improve care for PwP through remotely monitoring symptoms using wearable technology, increasing PD education in patients, encouraging self-management, and providing a healthcare contact (28). A recent feasibility analysis of this pathway lead to more patients feeling listened to and supported, key factors in improving self-management and medication adherence (28).

### 4.3 Survey limitations

This survey highlighted a common challenge often observed in survey research: respondents providing inconsistent answers to questions which should capture the same information. For example, only 37 respondents reported to using a smartwatch when asked which device they currently used to provide medication reminders, but when specifically asked if they used a smartwatch 66 respondents reported to using one. This may have been a consequence of individuals using multiple devices to provide reminders, and only reporting the most commonly used device, or may be a consequence of question fatigue, recall bias, cognitive impairment (although we did not test for this), and/or the fact that questions were written without consulting PwP. To ensure terminology used in future surveys is appropriate and accessible, it is imperative that PwP are consulted when developing future surveys. Furthermore, future surveys should utilise more ‘yes/no’ questions.

Due to the descriptive nature of this work, causal interpretation and implications of confounding variables are beyond the scope of this work, meaning findings should be interpreted with caution and taken as exploratory insights. Additionally, due to the nature of this survey, there was no way to confirm a PD diagnosis, or how long individuals had been diagnosed for. However, due to the length of the survey; the substantial effort required to complete it; the lack of financial incentive; and the fact it was mainly shared via PD specific charities and LinkedIn, the likelihood of someone falsely completing the survey is reduced. Given the limited representation of non-UK respondents (*n* = 11), these responses were excluded from the analysis, meaning study findings are only generalisable to UK-based PwP. As respondents were recruited through Parkinson's charities and professional networks the sample may not be representative of the wider population and observations should be interpreted with caution.

## 4 Conclusion

This survey provides exploratory insights to develop the ideal medication reminder technology for PwP. The survey observed that suboptimal medication adherence is common among UK-based PwP, but medication reminder technology may improve this. However, for this technology to be acceptable to PwP, it needs to be personalised to user preference and usability needs to be prioritised over aesthetics, with PwP included as co-developers during the design process. Additionally, utilisation of reminder technology alone may not sufficiently support medication adherence, and other factors which negatively influence adherence, such as mood disorders, need to be identified and overcome.

## Data Availability

The raw data supporting the conclusions of this article will be made available by the authors, without undue reservation.
