# Supplementary material for: Exploring medication experiences, behaviours, and reminder technology preferences in people with Parkinson's disease: a survey
Source: Front Digit Health. 2026 Jul 30;8:1839031. doi: 10.3389/fdgth.2026.1839031 (PMC13467758; doi:10.3389/fdgth.2026.1839031)
Supplement: Supplementary file 1 [file Datasheet1.docx]

# 1 Method

## 1.1 Survey design

The survey was designed as an open survey, meaning any individuals who met the inclusion criteria could access and take part in the survey. The survey was administered online using JISC Online Survey, a specialist survey website, which individuals only visit to fill out specific surveys and questionnaires. On March 7^th^, 2023, we added the question “Have you had a deep brain stimulation?”, meaning the first fourteen participants did not answer this question. Due to the substantial effort required to complete the survey and no financial or other incentives offered, and it was believed that false representation was unlikely, and therefore PD diagnosis was not verified.

Questions were ordered to avoid biasing answers to future questions. The number of items per page ranged from 1 to 9, excluding conditional questions and the survey was distributed over 46 pages. Respondents were able to review and change their answers by navigating to the previous pages. To reduce confusion, questions were conditionally displayed based on survey responses. Respondents were able to review and change their answers by navigating to the previous pages using the “Previous” button on the bottom left of each page. The survey did not collect the IP address of any individuals who took part in the survey.

## 1.2 Survey development

Between December 2022 and February 2023, PD experts pilot tested the survey, and provided feedback on survey usability (navigation, order of questions to avoid prior knowledge bias etc.), technical feasibility (display of conditional and mandatory questions etc.) and wording (use of technical jargon, appropriate wording for PwP etc). The final document was assessed by the whole team before its launch. A flyer was created and shared via social media (Supplementary Material).

## 1.3 Data Handling

To detect duplicate responses, respondents’ postcodes were screened. When identical postcodes were detected, respondents’ age and sex were compared, and if they matched (for age within 1 year of first response), the second set of data was removed, as this may have been biased by prior knowledge of questions. Age, sex and region of residence were compared when postcode was not provided. To anonymise responses, all respondents were given ID’s based on when they completed the survey – the first respondent was 1, the second 2 and so on.

## 1.4 Questions

To provide informed consent, respondents clicked Yes, I would like to complete this survey. Questionnaires which were terminated early were not included in analysis, only questions in which all questions had been responded to, or ‘prefer not to say’ was chosen were analysed. All complete questionnaires were analysed, and no cut-off point. Respondents who started the survey could leave at any point, and did not have to finish the survey.

## 1.5 Response categorising

To minimise misunderstanding and avoid misclassifying responses from open text responses, any answers that did not clearly fit into predefined categorises were reported exactly as written. Comorbidities were also reported as written by respondents, and only those which fell within the National Institute for Health and Care Excellence (NICE) definition of comorbidity were included. Responses with little detail or no diagnosis were not included, for example “shoulder pain”, “mental health”, “ankle has restricted movement”, or “nerve pain”. To avoid misinterpreting results, if respondents reported comorbidities using an acronym they were not included unless commonly used such as ADHD, whereas acronyms such as ET make reference to a number of conditions and were not included. Cramps and dysphagia were not included in the comorbidity list as these are common symptoms of PD.

# 2 Results

## 2.1 Response rate

A total of 2,019 entrees to the survey were recorded. Among these, 1,611 potential respondents stopped at the first page (participant information sheet), 46 did not continue past the informed consent question and 362 started the survey, giving a recruitment rate of 17.9%. Three hundred and nine individuals (85.4%) completed the survey. Average time to complete the survey was 29 minutes and 20 seconds.

Due to low responses from non-UK respondents (n=11), these respondents were removed from analysis. Of these respondents, three were from the United States of America, two from Ireland, and one respondent was from each of the following countries, Australia, Belgium, Canada, Germany, Israel, and Uganda.

## 2.2 Demographic characteristics

Of the 283 respondents, 87% were from England, 7% from Scotland, 4% from Wales, and 2% from Northern Ireland (Supplementary Table 1).

*Supplementary Table 1. Number of respondents from across the United Kingdom.*

| **UK Region** | **Number of respondents** |
| --- | --- |
| **England** |  |
| East Midlands | 16 |
| East of England | 30 |
| London | 21 |
| North East England | 32 |
| North West England | 19 |
| South East England | 49 |
| South West England | 37 |
| West Midlands | 22 |
| Yorkshire and the Humber | 19 |
| **Scotland** |  |
| Central Scotland | 7 |
| Glasgow | 3 |
| Highlands and Islands | 3 |
| Lothian | 2 |
| Mid Scotland and Fife | 2 |
| North East Scotland | 2 |
| South Scotland | 1 |
| West Scotland | 1 |
| **Wales** |  |
| South East Wales | 5 |
| South West Wales | 5 |
| **Northern Ireland** |  |
| Antrim | 2 |
| Armagh | 1 |
| Derry | 2 |
| County Down | 2 |

## 2.3 Quality of Life

Quality of life was assessed using the PDQ-8 questionnaire, which asks 8 questions about how often in the last month individuals experienced different factors. Responses highlighted that, ‘in the last month’ 20% of respondents often or always experienced painful muscle cramps or spasms, and 13% often or always experienced difficulty concentrating and getting around in public. Whereas, respondents experienced the least problems with close personal relationships, with 55% never experiencing this, and only 27% occasionally experiencing this (Supplementary Figure 1).

Supplementary Figure 1. Percentage of respondents and their responses to each question of the PDQ-8 questionnaire

Percentage of respondents

|  | Never | Occasionally | Sometimes | Often | Always |
| --- | --- | --- | --- | --- | --- |
| 1. Had difficulty getting around in public? | 44.5578 | 27.551 | 11.5646 | 8.84354 | 3.7415 |
| 2. Had difficulty dressing yourself? | 44.5578 | 29.5918 | 10.5442 | 8.84354 | 2.72109 |
| 3. Felt depressed? | 32.6531 | 37.415 | 16.6667 | 9.18367 | 0.340136 |
| 4. Had problems with your close personal relationships? | 55.4422 | 26.5306 | 9.86395 | 3.7415 | 0.680272 |
| 5. Had problems with your concentration, e.g. when reading or watching TV? | 28.9116 | 40.1361 | 14.6259 | 11.9048 | 0.680272 |
| 6. Felt unable to communicate with people properly? | 36.0544 | 38.4354 | 13.9456 | 7.14286 | 0.680272 |
| 7. Had painful muscle cramps or spasms? | 25.1701 | 33.3333 | 18.3673 | 18.7075 | 0.680272 |
| 8. Felt embarassed in public due to having Parkinson's disease? | 39.7959 | 36.7347 | 10.2041 | 7.82313 | 1.70068 |

## 2.4 Physical activity

Of the 254 respondents who were physically active, the majority took part in ‘walking outside’ (93%), 81% performed ‘household tasks’, 55% took part in ‘gardening’, and 52% in exercise classes (Supplementary Figure 2). Water based activities included swimming (n=7), aqua aerobics (n=2), underwater hockey and scuba diving (n=1). Thirteen respondents reported ‘other’ less physically active activities, these included: singing (n=4), knitting/sewing (n=3), family time (n=2), playing a musical instrument (n=2), crafts (n=2), photography (n=1), writing (n=1), cooking (n=1), and shopping (n=1). Family time was categorised from respondent quotes “looking after grandchildren”, and “running around looking after 3 active grandsons”. One respondent each took part in the following: bowels, boxing, physio, walking cricket, home exercises, and working as a hospital porter.

| Physical Activities | Percentage of physically active respondents |
| --- | --- |
| Walking outside | 92.9134 |
| Household tasks | 80.7087 |
| Gardening | 55.1181 |
| Exercise classes | 51.9685 |
| Strength training | 25.5906 |
| DIY | 24.8031 |
| Cycling | 18.1102 |
| Jogging | 5.51181 |
| Tennis/Badminton | 5.51181 |
| Pilates/Yoga | 4.33071 |
| Golf | 4.33071 |
| Water based activities | 3.93701 |
| Running | 3.14961 |
| Exercise Machines | 2.3622 |
| Gym | 1.9685 |
| Dance | 1.9685 |
| Table Tennis | 1.9685 |
| Football/walking football | 1.5748 |
| Martial Arts | 1.5748 |

Supplementary Figure 2. Percentage of physically active respondents and their physical activities.

## 2.5 PD symptoms

Respondents reported a median of 4 (1-9) PD symptoms. Twenty-six ‘other’ symptoms were reported. Most commonly, dystonia, cramps, and constipation were experienced by four respondents each (Supplementary Figure 3). We cannot determine if the reported symptoms are specific to PD, or are a side effect of medication, therefore we have reported ‘symptoms’ as reported by respondents, and not changed or regrouped, to reduce misreporting.

| 'Other' PD symptoms | Number of respondents |
| --- | --- |
| Dystonia | 4 |
| Cramps | 4 |
| Constipation | 4 |
| Apathy | 2 |
| Rigidity | 2 |
| Insomnia | 1 |
| Overactive bladder | 1 |
| Incontinence | 1 |
| Ageusia | 1 |
| Toe walking | 1 |
| Dribbling | 1 |
| Dry Mouth | 1 |
| Low mood | 1 |
| Insomnia | 1 |
| Brain fog | 1 |
| Anxiety | 1 |
| Diarrhoea | 1 |
| Increased urination | 1 |
| Joint swelling | 1 |
| Loss of dexterity | 1 |
| Fumbling fingers | 1 |
| Dehydration | 1 |
| Hot/cold temperature intolerance | 1 |
| Concentration difficulties | 1 |
| Hypotension | 1 |
| Reduced sleep | 1 |

Supplementary Figure 3. Number of respondents reporting 'other' symptoms.

### 2.5.1 PD symptom frequency and intensity

Among survey respondents, each PD symptom was experienced at different intensities and frequencies in the seven-days prior to completing the survey. In comparison to the other PD symptoms, freezing of gait was commonly reported at a severe intensity level (22%), but was also the symptom least likely to be experienced ‘always’. Whereas speech problems were least often reported at a severe intensity, but were experienced sometimes or often in the majority of respondents (82.1%) sometimes experiencing it (Supplementary Figure 4 and 5).

Percentage of respondents

| Symptom Intensity | Very mild | Mild | Moderate | Severe |
| --- | --- | --- | --- | --- |
| Tremor | 15.0538 | 39.7849 | 40.8602 | 4.30108 |
| Dyskinesia | 19.1304 | 39.1304 | 36.5217 | 5.21739 |
| Bradykinesia | 8.63636 | 41.8182 | 45.4545 | 4.09091 |
| Pain | 4.13793 | 27.5862 | 57.2414 | 10.3448 |
| Sleep Problems | 4.61538 | 29.2308 | 59.4872 | 6.66667 |
| Speech problems | 10.7143 | 55.9524 | 32.1429 | 1.19048 |
| Balance problems | 14.0496 | 42.9752 | 33.0579 | 9.91736 |
| Freezing of Gait | 11.7647 | 31.3725 | 35.2941 | 21.5686 |
| Symptom fluctuation | 4.23729 | 38.9831 | 44.0678 | 12.7119 |

Supplementary Figure 4. Percentage of respondents and how severe they felt each symptom was.

Percentage of respondents

| Symptom Frequency | Rarely | Sometimes | Often | Always | Prefer not to say |
| --- | --- | --- | --- | --- | --- |
| Tremor | 8.60215 | 29.5699 | 43.5484 | 18.2796 | 0 |
| Dyskinesia | 12.1739 | 50.4348 | 30.4348 | 6.95652 | 0 |
| Bradykinesia | 5.45455 | 38.6364 | 40.4545 | 15.4545 | 0 |
| Pain | 2.06897 | 31.0345 | 52.4138 | 13.7931 | 0.689655 |
| Sleep Problems | 2.05128 | 33.8462 | 48.2051 | 15.8974 | 0 |
| Speech problems | 5.95238 | 47.619 | 34.5238 | 11.9048 | 0 |
| Balance problems | 13.2231 | 47.9339 | 19.8347 | 19.0083 | 0 |
| Freezing of Gait | 13.7255 | 43.1373 | 39.2157 | 3.92157 | 0 |
| Symptom fluctuation | 4.23729 | 46.6102 | 37.2881 | 11.8644 | 0 |

Supplementary Figure 5. Percentage of respondents and how frequently they experienced each symptom.

### 2.5.2 Comorbidities

76 different comorbidities were reported, with arthritis most commonly reported, followed by high blood pressure, asthma and diabetes (Supplementary Table 2). From comorbidity 31 in table 2, all comorbidities were reported by one respondent.

Supplementary Table 2. Comorbidities which respondents experienced and the number of respondents who experienced each comorbidity.

| **Comorbidity** | **n** |
| --- | --- |
| Arthritis (osteo, psoriatic, rheumatoid) | 24 |
| High Blood Pressure | 19 |
| Asthma | 13 |
| Diabetes (Type 1 and 2) | 11 |
| Overactive Thyroid | 9 |
| Atrial Fibrillation | 7 |
| Osteoporosis | 7 |
| Hearing loss/deaf | 7 |
| Cancer (breast, prostate, skin, bladder, liver) | 7 |
| Depression | 6 |
| Hypercholesterolemia | 5 |
| Anxiety | 4 |
| Gastro-oesophageal reflux disease | 3 |
| Sleep apnoea | 3 |
| Sciatica | 3 |
| Chronic Obstructive Pulmonary Disease | 3 |
| Glaucoma | 3 |
| Hernia | 3 |
| Raynaud’s | 2 |
| Enlarged prostate | 2 |
| Heart disease | 2 |
| Macular degeneration | 2 |
| Deep vein thrombosis | 2 |
| Spinal stenosis | 2 |
| Fibromyalgia | 2 |
| Dementia | 2 |
| Kidney stones | 2 |
| Bipolar disorder | 2 |
| Lymphocytic Colitis | 2 |
| Hashimoto's thyroiditis | 2 |
| Irritable Bowel Disease | 2 |
| Coeliac | 1 |
| Acid reflux | 1 |
| Ulcerative Colitis | 1 |
| Overactive bladder | 1 |
| Heart disease | 1 |
| Vasculitis | 1 |
| Hypogonadism | 1 |
| Hypopituitarism | 1 |
| Insomnia | 1 |
| Goitre | 1 |
| Gout | 1 |
| Nephrotic syndrome | 1 |
| Psoriasis | 1 |
| Laryngeal reflux | 1 |
| Attention Deficit Hyperactivity Disorder | 1 |
| Ectatic Aorta | 1 |
| monoclonal gammopathy of unknown significance (MGUS) | 1 |
| Non-alcoholic fatty liver disease | 1 |
| Peripheral neuropathy | 1 |
| Oesophageal dysmotility | 1 |
| Epilepsy | 1 |
| Rhinitis | 1 |
| Dysphagia | 1 |
| Thyroid disease | 1 |
| Inactive diaphragm | 1 |
| Aspergers | 1 |
| Scoliosis | 1 |
| Irritable Bowel Syndrome | 1 |
| Prediabetic | 1 |
| Migraines | 1 |
| Plantar fasciitis | 1 |
| Angina | 1 |
| Thyrotoxicosis | 1 |
| Bullous pemphigoid | 1 |
| Functional movement disorder | 1 |
| Rapid Eye Movement Sleep Behaviour disorder | 1 |
| Brain aneurysm | 1 |
| Trigeminal neuralgia | 1 |
| Aortic dissection | 1 |
| Bursitis | 1 |
| Ventricular tachycardia | 1 |
| Lichen sclerosis | 1 |
| Gilbert syndrome | 1 |
| Osteogenesis imperfecta | 1 |
| Spinal Fracture | 1 |

### 2.5.3 PD medication

Supplementary figure 6 summarises the percentage of respondents who took their PD medication at the different times across a day.

Supplementary Figure 6. Percentage of respondents taking medication across all time points in the day.

| Medication Intake Time | Percentage of Respondents |
| --- | --- |
| Midnight | 1.76678 |
| 01:00:00 | 1.06007 |
| 02:00:00 | 0 |
| 03:00:00 | 1.06007 |
| 04:00:00 | 1.41343 |
| 05:00:00 | 2.12014 |
| 06:00:00 | 16.6078 |
| 07:00:00 | 38.8693 |
| 08:00:00 | 41.3428 |
| 09:00:00 | 15.5477 |
| 10:00:00 | 14.841 |
| 11:00:00 | 26.1484 |
| 12:00:00 | 37.1025 |
| 13:00:00 | 18.0212 |
| 14:00:00 | 24.735 |
| 15:00:00 | 18.0212 |
| 16:00:00 | 26.8551 |
| 17:00:00 | 25.4417 |
| 18:00:00 | 24.735 |
| 19:00:00 | 21.9081 |
| 20:00:00 | 21.9081 |
| 21:00:00 | 13.4276 |
| 22:00:00 | 30.0353 |
| 23:00:00 | 14.4876 |

## 2.6 Medication beliefs

Medication beliefs were assessed using the medication beliefs scale (PD-Rx). Overall, most respondents had positive beliefs concerning their PD medication: 69% of respondents agreed that their PD medication improves how fast they move, 79% felt that their PD medication helps them live a more normal life, and 79% felt that their PD medication gives them some control over their PD, with only 7% disagreeing with this. Additionally, 66% felt their PD medication makes walking easier. Negative beliefs were stronger than positive belief for 3 items: 63% of respondents agreed that their PD medication will lead to higher and higher doses in the future, 23% of respondents agreed that their PD medication will cause serious side effects in the future whereas 21% disagreed with this statement, and only 27% of respondents agreed that their PD medication slows the progression of their PD – which is true, and should therefore not be considered when exploring negative medication beliefs in PwP.

Supplementary Figure 7. Percentage of respondents with each response to the PD-Rx questions.

Percentage of respondents

| PD-Rx Questions | Strongly Disagree | Disagree | Neutral | Agree | Strong Agree |
| --- | --- | --- | --- | --- | --- |
| 1. Improves how fast or easily I move | 4.947 | 6.71378 | 19.4346 | 43.1095 | 25.7951 |
| 2. Causes extra unwanted movements | 19.0813 | 28.9753 | 28.2686 | 20.1413 | 3.53357 |
| 3. Makes walking easier | 4.59364 | 9.54064 | 19.788 | 43.8163 | 22.2615 |
| 4. Gives me bad side effects now | 30.3887 | 31.8021 | 16.6078 | 17.3145 | 3.88693 |
| 5. Improves my mood | 7.06714 | 12.3675 | 48.4099 | 23.6749 | 8.48057 |
| 6. Is a burden that disrupts my daily routine | 21.5548 | 32.1555 | 26.8551 | 16.6078 | 2.82686 |
| 7. Helps me live a more normal life | 2.12014 | 5.65371 | 13.7809 | 45.583 | 32.8622 |
| 8. Will lead to higher and higher doses in the future | 2.82686 | 3.88693 | 31.0954 | 46.6431 | 15.5477 |
| 9. Gives me some control over my PD | 1.41343 | 6.00707 | 13.0742 | 56.1837 | 23.3216 |
| 10. Slows the progression of my PD | 16.6078 | 22.2615 | 33.5689 | 21.2014 | 6.36042 |
| 11. Will cause serious side effects in the future | 4.59364 | 16.6078 | 56.1837 | 18.0212 | 4.59364 |

## 2.7 Parkinson’s specialist provided enough information?

Supplementary Table 3 summarises all responses to the question ‘Has your Parkinson's specialist given you information about the importance of taking your medication as prescribed?’. Responses were grouped into three major themes by EP and HD. Medication Timing, Medication Effect, and Food Effects, with other responses written into text to provide deeper insights.

Supplementary Table 3. Responses and theme groupings for question 27 of the survey.

| **Respondent ID** | **27. Response Yes/No** | **27.a. If No, and you are happy to provide more detail, please tell us in the text box below, what additional information you would have liked to receive: (Respondent Answers)** | **Theme/Comments** |
| --- | --- | --- | --- |
| CiC_S_31 | No | No more information needed. I read the leaflet inside the medication | Got more info from leaflet |
| CiC_S_45 | No | The impact of forgetting to take medication & how to juggle it when travelling across different time zones. | Medication Effect, Medication Timing |
| CiC_S_54 | No | Given verbal advice, which is fine | NA |
| CiC_S_92 | No | How medication can be affected by food. Why I can't take slow release madopar during the day. | Food effects, Medication Effect |
| CiC_S_99 | No | Importance, or not, of timing (time of day/gap between doses, and also in relation to meals and especially protein intake). | Medication Timing |
| CiC_S_112 | No | Best timings of medication. Whether you should alter medication related to the amount of exercise. Timings between medication should they be different day and night? | Medication Timing, Exercise |
| CiC_S_118 | No | what is likely to happen if I take more or less medication | Medication Effect |
| CiC_S_121 | No | I had to find out for myself about the timing of medication related to when you eat | Medication Timing |
| CiC_S_128 | No | I got the information I needed from the leaflets that came with the meds and from talking to Parkinson's UK helpline. | Got more info from leaflet, Parkinson's Helpline |
| CiC_S_133 | No | At what intervals. Pre or post food. Had to research myself. | Medication Timings |
| CiC_S_140 | No | When during the day to take it, avoiding certain foods, wearing off, optimisation of taking schedule. | Medication Timing, Food Effects, Wearing Off |
| CiC_S_141 | No | The importance of taking medication on time | Medication Timing |
| CiC_S_208 | No | Side effects, how long to take them, who will review them, what if they don't work. What if the side effects are too much. | Medication Effect, Medication Timing, Appointments |
| CiC_S_217 | No | to be honest i do not remember | NA |
| CiC_S_230 | No | any information. I was just prescribed the pills when I was in hospital and that was it. | Wanted 'any information', just prescribed and that was it |
| CiC_S_234 | No | If I dont take the medication what happens. Medication best practice. Take on an empty stomach. dont have with dairy etc. What effects the medication working | Food effects, Medication Effect |
| CIC_S_246 | No | advice on times to take dose though this is on medication label but specialist hasn't actually said during consultations | Medication Timing |
| CIC_S_250 | No | Timing and mixing with other prescribed drugs | Medication Timing |
| CIC_S_256 | No | communication from specialist is very limited/selective | Not given enough information |
| CIC_S_261 | No | Best times to take medication. How the medication works (i.e. in the gut) How long the dose can be expected to last - How to recognise when the medication is wearing off (and what "normal" looks like. Things that inhibit effectiveness of medication. (e.g. Protein) | Medication Timing, Medication Effect, Food Effects |
| CIC_S_268 | No | He just prescribed the tablets and that was that. | Just prescribed medication and that was it |
| CIC_S_284 | No | A tolerance to the given time, for example +/- 5 mins no problem, +/- 15 min OK, +/- 30 mins pushing it... | Medication Timing |
| CIC_S_297 | No | I have tweaked things myself and kept a journal for cause and effect | NA |
| CIC_S_299 | No | I saw a neurologist once at diagnosis given medication and told he would see me again in 2-3 months now told can’t see anyone for another 52 weeks as too many to see | Infrequent appointments |

## 2.8 Medication side effects

In the seven days prior to completing the survey, 16% (n=45) of respondents experienced medication side effects other than dyskinesia and wearing off, with nausea most commonly reported (n=11), followed by tiredness/fatigue (n=6), and dystonia (n=5). Whereas, when respondents considered medication side effects they had ‘ever’ experienced since taking PD medication, 28% of respondents (n=78) experienced side effects (other than dyskinesia and wearing off), with nausea most commonly reported (n=22), followed by dystonia (n=9) (directly reported by respondent or recategorised from reports of cramping or twisting), and tiredness/fatigue (n=8). Full details of all medication side effects are reported in supplementary table 4.

### 2.8.1 Dyskinesia and motor symptom fluctuations

Over the 7-days prior to completing the survey, 55% (n=154) (n=154, 88 male and 66 female) experienced dyskinesia, with 29% often or always experiencing it. Of the 154 respondents who experienced dyskinesia 30% agreed or strongly agreed that dyskinesia stopped them from completing their activities of daily living (Figure 2), and 76% agreed or strongly agreed that avoiding dyskinesia was important to them. Respondents with dyskinesia had a significantly higher QoL (PDQ-8) score (29 vs 19) (U=6506, *p*<0.001) and a significantly longer disease duration (U=6907, p<0.001) than those without dyskinesia.

Whereas, over the 7-days prior to completing the survey, 69% percent of respondents experienced motor symptom fluctuations (wearing off), with 23% often or always experiencing them. Forty-eight percent of respondents who experienced fluctuations agreed or strongly agreed that they stop them from completing activities of daily living and 80% agreed or strongly agreed that avoiding fluctuations was important to them (Figure 2).

Percentage of respondents

|  | Strongly disagree | Disagree | Neutral | Agree | Strongly agree | Not applicable |
| --- | --- | --- | --- | --- | --- | --- |
| Avoiding motor fluctuations is important to me | 9.74359 | 20.5128 | 16.4103 | 37.9487 | 10.2564 | 5.12821 |
| Motor fluctations stop me from completing my activites of daily living | 3.07692 | 5.12821 | 10.2564 | 37.4359 | 42.5641 | 1.02564 |
| Avoiding dyskinesia is important for me | 2.5974 | 5.19481 | 14.9351 | 48.7013 | 27.2727 | 0.649351 |
| Dyskinesia stops me from completing my daily activities | 14.2857 | 33.7662 | 18.8312 | 25.3247 | 5.19481 | 2.5974 |

Figure 8. Percentage of respondents with motor symptom fluctuations and dyskinesia, and their agreement to four statements.

Supplementary Table 4 summarises all side effects reported by respondents ‘ever’ and in the ‘seved-days prior to completing the survey. Five respondents did not report which side effects they experienced, but were kept in analysis. However, 14 respondents only reported dyskinesia and were excluded, with an additional 2 respondents reporting ‘wearing off’ in the seven day question and were excluded and one respondent just reporting ‘tremor’ and was further excluded from this question.

Supplementary Table 4. Reported medication side effects that respondents experienced 'ever' since taking their medication, and in the 7-days prior to completing the survey.

| **Medication Side Effect** | **Ever (n)** | **7-Days Prior to completing survey (n)** |
| --- | --- | --- |
| Nausea | 22 | 11 |
| Dystonia | 9 | 5 |
| Tiredness/Fatigue | 8 | 6 |
| Impulsive/Compulsive Behaviour | 6 | 2 |
| Constipation | 5 | 4 |
| Sleepiness/Drowsiness | 5 | 4 |
| Sleep Issues/Disturbances | 5 | 3 |
| Hypo/hypertension | 5 | 2 |
| Dry mouth | 5 | 1 |
| Hallucinations/Delusions/Paranoia | 4 | 2 |
| Dizziness | 4 | 1 |
| Headaches | 3 | 4 |
| Diarrhoea | 3 | 0 |
| Pain | 2 | 2 |
| Increased balance problems | 2 | 1 |
| Sweating | 2 | 1 |
| Stomach Problems | 2 | 0 |
| Vomiting | 2 | 0 |
| Decreased labido | 1 | 1 |
| Anxiety | 1 | 2 |
| More severe tremor | 1 | 2 |
| Memory problems | 1 | 2 |
| Oedema | 1 | 2 |
| Skin reactions | 1 | 1 |
| Sexual Dysfunction | 1 | 1 |
| Increased urination | 1 | 1 |
| Heart palpitations | 1 | 1 |
| Feeling faint | 1 | 0 |
| Indigestion | 1 | 0 |
| Rhinitis | 1 | 0 |
| Breathing dysregulation | 1 | 0 |
| Suicidal Thoughts | 1 | 0 |
| Restless legs | 1 | 0 |
| Heartburn | 1 | 0 |
| Brain Fog | 0 | 5 |
| Rigidity | 0 | 2 |
| Short tempered | 0 | 1 |
| Neuropathy | 0 | 1 |
| Problems with speech | 0 | 1 |
| Catatonia | 0 | 1 |

## 2.9 Medication reminder technology

Supplementary Table 5. Responses from respondents who said they would not use a digital device to remind them to take their medication, and the groupings.

|  | **39. Would you use a digital device to remind you to take your medication (e.g., automatic tablet dispenser, smartphone etc.) ?** | **39.b. If No, and you are happy to provide more information, please let us know in the text box below why you would not use such a device.** | **Grouping** |
| --- | --- | --- | --- |
| CiC_S_03 | No | Prefer to do it myself | Does not feel the need |
| CiC_S_11 | No | Take next med when needed | Does not follow a set medication routine |
| CiC_S_30 | No | I have enough trouble with fiddly things without introducing something else. I live on my own and I do not wish anybody to have to come around and put something on me. I value my medical privacy and do not wish to use anything that necessated by somebody else to share my medical information. Tech is hard for people with Parkinson's - I really resent the introduction of things that mean people with Parkinson's become more reliant on family and friends instead of maintaining independence. I have no faith whatsoever in the privacy policies of such devices and associated apps. | Privacy and Independence concerns |
| CiC_S_55 | No | I do not need to be reminded. | Does not feel the need |
| CiC_S_80 | No | I can remember when to take my medication | Does not feel the need |
| CiC_S_97 | No | It’s not necessary at the moment. I am still feeling in charge. | Does not feel the need |
| CiC_S_117 | No | I don't need this at present.. | Does not feel the need |
| CiC_S_139 | No | At the moment I can take my medication as I feel it wearing of so I know that i have to take my medication | Does not follow a set routine |
| CiC_S_230 | No | not into using technology | Digital literacy issues |
| CIC_S_239 | No | Use pill box. | Uses other device - does not feel the need |
| CIC_S_287 | No | find tech confusing | Digital literacy issues |

| *Supplementary Table 6. CHERRIES Checklist* | | | |
| --- | --- | --- | --- |
| **Item Category** | **Checklist Item** | **Explanation** | **Page Number** |
| **Design** | Describe survey design | Describe target population, sample frame. Is the sample a convenience sample? (In “open” surveys this is most likely.) | Page 3 and 4, and Supp Material Page 1 |
| **IRB (Institutional Review Board) approval and informed consent process** | IRB approval | Mention whether the study has been approved by an IRB. | Page 3 |
|  | Informed consent | Describe the informed consent process. Where were the participants told the length of time of the survey, which data were stored and where and for how long, who the investigator was, and the purpose of the study? | Page 4 and supplementary material |
|  | Data protection | If any personal information was collected or stored, describe what mechanisms were used to protect unauthorized access. | Supp Material Page 1 |
| **Development and pre-testing** | Development and testing | State how the survey was developed, including whether the usability and technical functionality of the electronic questionnaire had been tested before fielding the questionnaire. | Supp Material Page 1 |
| **Recruitment process and description of the sample having access to the questionnaire** | Open survey versus closed survey | An “open survey” is a survey open for each visitor of a site, while a closed survey is only open to a sample which the investigator knows (password-protected survey). | Page 3 |
|  | Contact mode | Indicate whether or not the initial contact with the potential participants was made on the Internet. (Investigators may also send out questionnaires by mail and allow for Web-based data entry.) | Page 3/4 |
|  | Advertising the survey | How/where was the survey announced or advertised? Some examples are offline media (newspapers), or online (mailing lists – If yes, which ones?) or banner ads (Where were these banner ads posted and what did they look like?). It is important to know the wording of the announcement as it will heavily influence who chooses to participate. Ideally the survey announcement should be published as an appendix. | Page 3/4 |
| **Survey administration** | Web/E-mail | State the type of e-survey (eg, one posted on a Web site, or one sent out through e-mail). If it is an e-mail survey, were the responses entered manually into a database, or was there an automatic method for capturing responses? | Supp Material Page 1 |
|  | Context | Describe the Web site (for mailing list/newsgroup) in which the survey was posted. What is the Web site about, who is visiting it, what are visitors normally looking for? Discuss to what degree the content of the Web site could pre-select the sample or influence the results. For example, a survey about vaccination on a anti-immunization Web site will have different results from a Web survey conducted on a government Web site | Supp Material Page 1 |
|  | Mandatory/voluntary | Was it a mandatory survey to be filled in by every visitor who wanted to enter the Web site, or was it a voluntary survey? | Page 4, Supp Material Page 1 |
|  | Incentives | Were any incentives offered (eg, monetary, prizes, or non-monetary incentives such as an offer to provide the survey results)? | Supp Material Page 1 |
|  | Time/Date | In what timeframe were the data collected? | Page 3 |
|  | Randomization of items or questionnaires | To prevent biases items can be randomized or alternated. | Supp Material Page 1 |
|  | Adaptive questioning | Use adaptive questioning (certain items, or only conditionally displayed based on responses to other items) to reduce number and complexity of the questions. | Page 4 and Supp Material Page 1 |
|  | Number of Items | What was the number of questionnaire items per page? The number of items is an important factor for the completion rate. | Supp Material Page 1 |
|  | Number of screens (pages) | Over how many pages was the questionnaire distributed? The number of items is an important factor for the completion rate. | Supp Material Page 1 |
|  | Completeness check | It is technically possible to do consistency or completeness checks before the questionnaire is submitted. Was this done, and if “yes”, how (usually JAVAScript)? An alternative is to check for completeness after the questionnaire has been submitted (and highlight mandatory items). If this has been done, it should be reported. All items should provide a non-response option such as “not applicable” or “rather not say”, and selection of one response option should be enforced. | Page 4 and Supp Material Page 1 |
|  | Review step | State whether respondents were able to review and change their answers (eg, through a Back button or a Review step which displays a summary of the responses and asks the respondents if they are correct). | Supp Material Page 1 |
| **Response Rates** | Unique site visitor | If you provide view rates or participation rates, you need to define how you determined a unique visitor. There are different techniques available, based on IP addresses or cookies or both. | N/A |
|  | View rate (Ratio of unique survey visitors/unique site visitors) | Requires counting unique visitors to the first page of the survey, divided by the number of unique site visitors (not page views!). It is not unusual to have view rates of less than 0.1 % if the survey is voluntary. | N/A |
|  | Participation rate (Ratio of unique visitors who agreed to participate/unique first survey page visitors) | Count the unique number of people who filled in the first survey page (or agreed to participate, for example by checking a checkbox), divided by visitors who visit the first page of the survey (or the informed consents page, if present). This can also be called “recruitment” rate. | Supp Material Page 2 |
|  | Completion rate (Ratio of users who finished the survey/users who agreed to participate) | The number of people submitting the last questionnaire page, divided by the number of people who agreed to participate (or submitted the first survey page). This is only relevant if there is a separate “informed consent” page or if the survey goes over several pages. This is a measure for attrition. Note that “completion” can involve leaving questionnaire items blank. This is not a measure for how completely questionnaires were filled in. (If you need a measure for this, use the word “completeness rate”.) | Supp Material Page 2 |
| **Preventing multiple entries from the same individual** | Cookies used | Indicate whether cookies were used to assign a unique user identifier to each client computer. If so, mention the page on which the cookie was set and read, and how long the cookie was valid. Were duplicate entries avoided by preventing users access to the survey twice; or were duplicate database entries having the same user ID eliminated before analysis? In the latter case, which entries were kept for analysis (eg, the first entry or the most recent)? | Supp Material Page 1  Duplicate section: page 4 |
|  | IP check | Indicate whether the IP address of the client computer was used to identify potential duplicate entries from the same user. If so, mention the period of time for which no two entries from the same IP address were allowed (eg, 24 hours). Were duplicate entries avoided by preventing users with the same IP address access to the survey twice; or were duplicate database entries having the same IP address within a given period of time eliminated before analysis? If the latter, which entries were kept for analysis (eg, the first entry or the most recent)? | Supp Material Page 1 |
|  | Log file analysis | Indicate whether other techniques to analyze the log file for identification of multiple entries were used. If so, please describe. | N/A |
|  | Registration | In “closed” (non-open) surveys, users need to login first and it is easier to prevent duplicate entries from the same user. Describe how this was done. For example, was the survey never displayed a second time once the user had filled it in, or was the username stored together with the survey results and later eliminated? If the latter, which entries were kept for analysis (eg, the first entry or the most recent)? | N/A |
| **Analysis** | Handling of incomplete questionnaires | Were only completed questionnaires analyzed? Were questionnaires which terminated early (where, for example, users did not go through all questionnaire pages) also analyzed? | Supp Material Page 1 |
|  | Questionnaires submitted with an atypical timestamp | Some investigators may measure the time people needed to fill in a questionnaire and exclude questionnaires that were submitted too soon. Specify the timeframe that was used as a cut-off point, and describe how this point was determined. | Supp Material Page 1 |
|  | Statistical correction | Indicate whether any methods such as weighting of items or propensity scores have been used to adjust for the non-representative sample; if so, please describe the methods. | N/A |
